# Cereals and cereal products – a scoping review for Nordic Nutrition Recommendations 2023

**DOI:** 10.29219/fnr.v68.10457

**Published:** 2024-02-14

**Authors:** Guri Skeie, Lars T. Fadnes

**Affiliations:** 1 Department of Community Medicine, UiT The Arctic University of Norway, Tromsø, Norway; 2 Department of Global Public Health and Primary Care, University of Bergen, Bergen, Norway

**Keywords:** cereals, whole grains, dietary fiber, dietary guidelines

## Abstract

Cereals and cereal products have traditionally been staple foods in many countries including in the Nordics and Baltics. Cereals can be consumed with their entire grain kernel and are then referred to as whole grains or can be consumed after removal of the bran or germ and are then referred to as refined grains. The terms cereals and grains are often used interchangeably. In this scoping review, we examine the associations between intake of cereals and cereal products and major health outcomes to contribute to up-to-date food-based dietary guidelines for the Nordic and Baltic countries in the Nordic Nutrition Recommendations 2023 project. Five qualified systematic reviews that covered non-communicable diseases, mortality, and risk factors were identified, and a supplementary literature search was performed in the MEDLINE and Cochrane databases for more recent studies and other endpoints. Compared to other high-income countries, the Nordic populations have a high consumption of whole grain foods. In some of the countries, rye constitutes a substantial fraction of the cereal consumption. However, few studies are available for specific cereals, and most of the research has been performed in predominantly wheat-consuming populations. The evidence suggests clear dose–response associations between a high intake of whole grains and lower risks of cardiovascular disease, colorectal cancer, type 2 diabetes, and premature mortality. The lowest risks of morbidity and mortality were observed for 3–7 servings of whole grains per day, equivalent of 90–210 g/day (fresh weight or ready-to-eat whole grain products, such as oatmeal or whole grain rye bread). Evidence from randomized trials indicates that a high intake of whole grains is beneficial for reducing weight gain. There is less evidence for refined grains, but the available evidence does not seem to indicate similar beneficial associations as for whole grains. It is suggested that replacing refined grains with whole grains would improve several important health outcomes. Cereals are plant foods that can be grown in most of the Nordic and Baltic regions.

## Popular scientific summary

- Cereals are staple foods in the Nordic and Baltic countries.
- Whole grains are good sources of dietary fiber and several vitamins and minerals like B vitamins, vitamin E, iron, magnesium, and selenium.
- Recent evidence suggests that a high intake of whole grains is associated with lower risk of cardiovascular disease, colorectal cancer, type 2 diabetes, and premature mortality.
- A high intake of whole grains is beneficial for reducing weight gain.
- There is limited evidence available on the effects of refined grains and specific cereals.

Cereals are cultivated grasses and members of the Poaceae family, and the most frequently consumed types in the Nordic and Baltic countries are wheat, rye, oats, rice, and barley. The terms ‘cereals’ and ‘grains’ are often used interchangeably, but strictly speaking, ‘cereals’ is a bit more limited term as it includes only the grasses. When studying the health effects of cereals (grains), pseudo-cereals such as buckwheat and amaranth are also frequently included in the culinary definitions. However, the bulk of the research has been done in predominantly wheat-consuming populations and less commonly on specified cereals (1, 2). Botanically, the cereal grains (kernels) are fruits, consisting of bran, endosperm, and germ. Cereals can be consumed with their entire grain kernel and are then referred to as whole grains or can be consumed after the removal of the bran or germ and are then referred to as refined grains. In a whole grain and in the whole grain part of a whole grain product, the bran, germ, and endosperm are present in the same proportion as in the natural state, although husk and hull have been removed (1, 3). In this scoping review, we refer to cereals as whole grains or refined grains to reflect which components of the grain have been kept, while non-cereal grains will not be covered. Many of the nutrients in grains are concentrated in the outer layer, the bran. In food production, the bran is often removed, creating various refined grain products. We will not review the health effects of bran or other grain components. Whole grains and refined grains have distinct health effects (see below). The same is the case for products based on whole grains and refined grains, and many refined grain products contain substantial amounts of added sugars.

While the definition of whole grains *per se* has generally reached a consensus, the definition of whole grain foods or products – and how much of the food that should be whole grain for it to be classified as a whole grain food – is still a matter for discussion. This complicates comparisons between studies, and different definitions give rise to different intake estimates (4–6). Lately, a definition saying that ‘whole-grain food shall contain at least 50% whole-grain ingredients based on dry weight’ has been endorsed by several organizations (1). It is suggested that foods with a minimum of 25% whole grain ingredients can be labeled with a front-of-pack nutrition claim but cannot have whole grain in its name (1). The Nordic Keyhole (front of pack label) can be added to food products that contain a certain amount of whole grain (food group dependent). The definition of whole grain is similar to the one used in this review, and wheat, spelt, rye, oats, barley, maize, rice, millet, durra, and other sorghum species are included (7). In the studies included in this review, no common definition of whole grains has been used.

Traditionally, grain products have been staple foods in many cultures, and they still are a dominating food group in many countries. With the improved economy, other food groups that were formerly mostly used in moderation now contribute to a larger proportion of the energy and nutrient intake in many populations, often at the expense of grain products. Still, grain products are important contributors to energy, carbohydrate, and protein intake and are key sources of dietary fiber, selenium, iron, a range of B-vitamins, and vitamin E (8, 9). The Global Burden of Disease study listed low intake of whole grain as a leading dietary risk factor, with 3 million deaths in 2017 attributable to this factor (10). However, the Nordic Nutrition Recommendations (NNR) 2012 did not give specific recommendations for cereals/grains although cereals were mentioned several times – most comprehensively in the systematic review on dietary patterns (11). However, most Nordic and Baltic countries have issued their own food-based dietary guidelines where cereal foods or foods with whole grains play an important part (12–18). In this scoping review, we describe the overall evidence for associations between consumption of grains (cereals), including whole and refined grains, and their associations with health-related outcomes as a basis for setting and updating food based dietary guidelines in the NNR2023 (Box 1).

*Box 1.* Background papers for Nordic Nutrition Recommendations 2023.

- This paper is one of many scoping reviews commissioned as part of the Nordic Nutrition Recommendations 2023 (NNR2023) project (19)
- The papers are included in the extended NNR2023 report but, for transparency, these scoping reviews are also published in Food & Nutrition Research
- The scoping reviews have been peer reviewed by independent experts in the research field according to the standard procedures of the journal
- The scoping reviews have also been subjected to public consultations (see report to be published by the NNR2023 project)
- The NNR2023 committee has served as the editorial board
- While these papers are a main fundament, the NNR2023 committee has the sole responsibility for setting dietary reference values in the NNR2023 project

## Methods

This scoping review follows the protocol developed within the NNR2023 project (19). The sources of evidence used in the review follow the eligibility criteria described in Christensen et al. (20). As it was not intended that the NNR2023 project should duplicate work, a search was done for qualified systematic reviews in PubMed/MEDLINE and by inspection of the websites of national and international food and health authorities (21). Major national food and health authorities and organizations were also contacted directly for information on previous or planned systematic reviews. No *de novo* systematic reviews with relevance for this review were commissioned as part of the NNR2023 project. From the list of qualified systematic reviews relevant for the NNR2023 project (21), five had relevance for the present review (22–26). These were led by the UK Scientific Advisory Committee on Nutrition (23), Reynolds et al. (24), Hauner et al. (22) developing evidence based dietary guidelines from the German Nutrition Society, Åkesson et al. (25) for NNR2012, and the World Cancer Research Fund (WCRF) as part of their continuous update project (26). The most recent was the paper by Reynolds et al., commissioned as part of WHO’s work on developing updated recommendations for carbohydrate intake (24). This paper has dose–response data for major outcomes and focussed on carbohydrate quality including whole grains and was therefore chosen as our main source of evidence. The results from the relevant qualified systematic reviews are emphasized but also supported by evidence from an additional literature search. The search strategy for the additional search was: (cereal*[Title] OR grain*[Title] OR ‘whole grain*’ [Title]) AND (‘2011’[Date – Publication] : ‘3000’[Date – Publication]) AND humans[Filter] AND review[Publication Type]). This was updated to also include meta-analyses: *(cereal[MeSH Terms] OR cereals[MeSH Terms] OR Edible Grain[MeSH Terms] OR whole grains[MeSH Terms]) AND humans[Filter] AND (systematic review[Publication Type] OR meta-analysis[Publication Type]*). Searches were performed in MEDLINE (January 26th 2022, 229 hits) and Cochrane Reviews (January 27^th^ 2022, 14 hits) databases.

As previously mentioned, servings of grains can be given with different measures. One serving of whole grain is often defined as 30 g ready-to-eat-weight whole grain products (such as Danish or German whole grain rye bread or oatmeal porridge) (2, 24). This can also be labeled as fresh-weight. For products such as pasta and rice, serving sizes are often much larger (2). For products that are not 100% whole grain, calculation of whole grain requires recipe information, and how and whether this is done seems to vary. One serving in dry weight corresponds to approximately 10 g (which, for example, could be dry oatmeal, dry barley grains, or whole grain flour).

## Dietary intake in Nordic and Baltic countries

The Nordic countries have a high intake of cereals and cereal products, particularly whole grains (27, 28). There is less documentation from the Baltic countries, and differences in methods used to collect dietary data make comparisons complicated (29). According to one source, the daily intake of whole grains products (fresh weight) was above 70 g/day in all the Nordic countries, while in the Baltic countries, the daily whole grain product intake was between 20 and 25 g/day (27). Assuming that the energy contribution from carbohydrates at least to some extent reflects grain intake, it seems that in the Baltic countries, the grain intake is somewhat lower than in the Nordic countries, particularly in Latvia and Lithuania. For example, the energy percentage from carbohydrates in Finnish women/men is 46/43, compared to 39/39 in women/men from Latvia (29). This, combined with lower per capita availability of whole grain products in the Baltic countries (27), suggests that there are some differences in consumption between the countries. It is also noteworthy that when expressed as energy percentage, carbohydrate intakes are higher among women than among men in all countries except Latvia, where intakes were similar between genders. Unfortunately, the eight countries do not define or report cereals and cereal sub-groups similarly, so comparison of cereal intake is difficult. For instance, there is no estimate of the total intake from Iceland and Estonia, and there are lacking data for one or more cereal subgroups for most countries (29). Based on the data available for cereals, intakes are lower in Finland and Iceland than the rest of the countries. Bread is the most frequently eaten cereal product but also porridge is an important source, particularly in Finland and Estonia. Breakfast cereals (which often include oats) are consumed in variable amounts, particularly in Denmark, Sweden, and Iceland, while *other grains* are also contributing to the cereal intake, particularly in Estonia, Latvia, and Norway (29). Some of this could be for example rice and pasta, but based on food supply data, rice is much less frequently consumed in the region compared to wheat and rye (28). A previous paper based on data from the Scandinavian countries found that bread was the major source for whole grains in Norway and Denmark, while breakfast cereals and crispbread/rusks were important sources in Sweden (30). Wheat was the main source of whole grains in Norway, while rye was the largest contributor to the whole grain intake in Sweden and Denmark.

## Health outcomes relevant for Nordic and Baltic countries

Grains (or cereals) is a heterogeneous food group, and whole grains and refined grains often have different associations with health outcomes. In the following, the focus is therefore on whole grains and where there is evidence also on refined grains.

Cardiovascular diseases and cancers are the leading causes of mortality in the Nordic and Baltic countries (31, 32). As summarized in the qualified systematic reviews (22–26), the evidence for the consumption of whole grains and a reduced risk of colorectal cancer incidence is moderate to strong (24, 26), while the evidence for coronary heart disease, and all-cause mortality is low, mainly because of some unexplained heterogeneity (24). In short, a high compared to low intake of whole grains is associated with an approximately 10–15% reduction in colorectal cancer incidence, and a 20% reduction in coronary heart disease and all-cause mortality (Table 1) (24, 26). These results relate to an intake up to 100 g whole grains products/day for all-cause mortality and up to 360 g/day for colorectal cancer (24). For some of the outcomes, the associations seem to be leveling off, while for colorectal cancer, the association was linear (24). Other authors have chosen to mainly look at intakes up to 225 g/day (2) and found similar results.

**Table 1:** Summary of association between whole grains and major health outcomes from observational studies, comparing highest and lowest intakes [Reynolds et al. (24), supplemented with evidence from WholEUGrain (33)]

Outcomes	Studies	Cases	Relative risk (95% CI)	GRADE^a^	Evidence^b^
All-cause mortality	9	99,224	0.81 (0.72–0.90)	Low	Convincing
Coronary heart disease mortality	2	1,588	0.66 (0.56–0.77)	Low	Convincing
Coronary heart disease incidence	6	7,697	0.80 (0.70–0.91)	Low	Convincing
Stroke mortality	2	694	0.74 (0.58–0.94)	Low	Limited
Stroke incidence	3	1,247	0.86 (0.61–1.21)	Very low	Limited
Type 2 diabetes incidence	8	14,686	0.67 (0.58–0.78)	Low	Convincing
Colorectal cancer incidence	7	8,803	0.87 (0.79–0.96)	Moderate	Probable
Cancer mortality	5	32,727	0.84 (0.76–0.92)	Low	Limited

a The GRADE system assess the methodological quality of the studies, from Table 2 in (24).

b The evidence is evaluated based on methodological quality & biological plausibility (33).

In general, the quality of the evidence was graded as low, except for colorectal cancer incidence (moderate to strong) and stroke incidence (very low). The low grading was mainly due to unexplained heterogeneity of the results (24). When biological plausibility and the totality of the evidence was assessed, most associations (except for stroke and cancer mortality) were judged to be convincing or probable (33).

The results of dose–response relationships showed a similar pattern as the high-low comparisons (Table 2) (24). Within the amounts of whole grain currently recommended, it is reasonable to assume a linear relationship between whole grain intake and a reduction in risk of the health outcomes described here (24).

**Table 2:** Summary of dose-response relationships per 15 g^a^ intake of whole grains and major health outcomes from observational studies [Reynolds et al. (24), from table C1]

Outcomes	Studies	Person years (million)	Relative risk (95% CI)
All-cause mortality	9	8.2	0.94 (0.92–0.95)
Coronary heart disease mortality	3	1.9	0.91 (0.89–0.94)
Coronary heart disease incidence	6	2.4	0.93 (0.89–0.98)
Stroke mortality	3	1.9	0.94 (0.90–0.98)
Stroke incidence	3	1.1	0.97 (0.92–1.02)
Type 2 diabetes incidence	7	3.5	0.88 (0.81–0.95)
Colorectal cancer incidence	8	5.7	0.97 (0.95–0.99)
Cancer mortality	7	7.6	0.95 (0.93–0.97)

a A whole grain intake of 15 g/day in fresh-weight/ready-to-eat-weight of whole grain products corresponds to half a serving per day. The corresponding serving size in dry weight is approximately 5 g/day.

## Mechanisms

Whole grains are important contributors to dietary intake of minerals such as iron, magnesium, phosphorus, zinc, copper, manganese, and selenium, vitamins such as thiamine, riboflavin, niacin, pantothenic acid, pyridoxin, biotin, and folate, and some grains are also rich in tocopherols (33). Whole grains and whole grain products are also rich in dietary fiber, which is fermented by the microbiota into short-chain fatty acids. The fiber component of whole grain foods plays an important role in the protective effects of whole grains, but numerous bioactive compounds are also assumed to contribute (34–36). Short-chain fatty acids have shown anti-proliferative effects in experimental studies, which may contribute to the inverse associations observed for colorectal cancers. The evidence for dietary fiber and reduction in the risk of colorectal cancer is reported as strong by the World Cancer Research Fund (26). This is supported by both human and animal studies, with anti-carcinogenic and anti-oxidative properties found in bran and germ of whole grains. Further, fiber can have anti-proliferative properties on colon cancer cells, reduces intestinal transfer time, and contributes to health-promoting changes in the microbiota composition (22). It has also been suggested that whole grains bind carcinogens and help regulate glycemic response, both properties beneficial in relation to colorectal cancer. Dietary fibers generally increase the viscosity and reduce the time of food passage through the colorectum. Reduced passage time and the dilution effect of fibers both contribute to reducing carcinogenic effects of other dietary mutagens (33). Further, whole grains contain lignans that can be considered the plant’s own natural defence systems, which might have important roles for the microbiota. Phenolic compounds in whole grains have antioxidative properties, while phytosterols and β-glucans (found in, for example, oats) are known for their cholesterol lowering effects. On the other hand, grains can contain unwanted substances and contaminants, such as mycotoxins, pesticides, and metalloids, but the use and content of these substances are regulated, and this generally limits their negative effect on public health (33). A new Swedish report evaluated the risks and benefits of whole grain consumption in Sweden (37). It was modeled on the proportion of whole grain products of total cereal intake and found that even if all the intake of cereal products was whole grain products, the benefits in terms of reducing incidence of first myocardial infarctions outweighed possible negative effects caused by cadmium and mycotoxins. Mean total cereal intake was 166/218 g per day for women/men and whole grain intake in the population was 34/40 g per day for women/men.

Relating to biomarkers for disease and intermediate markers of morbidity, evidence from randomized trials indicates that higher compared with lower intakes of whole grains are associated with a reduction in systolic blood pressure, total cholesterol, and body weight (Table 3) (24). The quality of the evidence from these trials was judged to be moderate according to the GRADE system. All these biomarkers are important risk factors and mediating mechanisms for cardiovascular disease. Further, a high intake of whole grains is also linked with lower glycated hemoglobin, but the evidence for this is slightly lower due to relatively few trials and the heterogeneity of results between the randomized trials.

**Table 3:** Summary of key health outcomes from randomized trials from Reynolds et al. (24)

Outcomes in randomized trials	Studies	Participants	Effect size (95% CI)^a^	Grade
Change in bodyweight (kg)	11	498	MD –0.62 (–1.19;–0.05)	Moderate
Change in glycated haemoglobin A1c (%)	3	141	SMD –0.54 (–1.28;0.20)	Low
Change in total cholesterol (mmol/L)	17	772	MD –0.09 (–0.23;0.04)	Moderate
Change in systolic blood pressure (mmHg)	8	493	MD –1.01 (–2.46;0.44)	Moderate

a MD, mean difference; SMD, standardised mean difference.

Dietary fiber from whole grains contribute to higher levels of satiation and improves the postprandial glycemic response (33). A systematic review and meta-analysis of randomized controlled trials of consumption of whole grains compared to refined grains showed that whole grains were linked with fullness and satiety, which could partly explain the that whole grains are associations between with less overweight and obesity in contrast to refined grains (38, 39).

The evidence for an association between intake of dietary fiber and reduced body weight seems to be slightly stronger than for intake of whole grains as a food group (24, 40). A high versus low intake of whole grains is associated with lower risk of overweight/obesity (RR 0.85; 95% confidence interval 0.79–0.91) in observational studies (and correspondingly per serving, RR 0.93; 0.89–0.96) (38). Similarly, a high compared to low intake of whole grains was associated with lower risk of >2 kg weight gain (RR 0.83; 0.70–0.97). Refined grains, in contrast, were not significantly associated with risk of overweight/obesity (RR 1.11; 0.85–1.45, and RR 1.05; 1.00–1.10 per serving). Similarly for high versus low intake of refined grains and weight gain >2 kg, there were no significant association (RR 1.05; 0.78–1.41). The trials aiming at change in body weight by increasing whole grain intake found a mean difference of –0.62 kg (95% CI –1.19 to –0.05) between high and low consumers. Both HbA1c and body weight differences could explain inverse associations with type 2 diabetes. Trials on biomarkers for disease and risk factors were generally of shorter duration and involved fewer participants than observational studies (24). All the above-mentioned factors could be potential effect mediators contributing to the risk-reducing associations reported for cardiovascular disease and all-cause mortality.

## Food-based dietary guidelines

When integrating observational evidence with evidence from randomized trials on intermediate markers and mechanisms, the evidence for protective effects from a high intake of whole grains related to all-cause mortality, cardiovascular disease and type 2 diabetes is considered convincing (33). There is less evidence for specific whole grain products, but both whole grain bread, breakfast cereals, and oats/oatmeal has also been associated with reduction in cardiovascular disease (41). Similar associations were found for all-cause mortality (2). The evidence for protective effects related to colorectal cancer are considered as probable. Umbrella reviews have provided similar results to what we have extracted from the systematic reviews (42). Current recommendations on whole grain intake aimed at adults are often related to energy intake, such as recommending 75 g of whole grains per 10 MJ, corresponding to around 250 g of ready-to-eat whole grain products (43). Infants and children have lower energy requirements, and whole grain recommendations should be scaled to reflect this. In a systematic review on dietary fiber intake in childhood or adolescence and subsequent health outcomes, no adverse effects were found from higher intakes of whole grains (44). Based on the totality of the evidence, even though the data from children is sparse, the authors suggest that it is reasonable to derive recommendations for children based on the results obtained for adults. When updating the Danish whole grain recommendations, several scenarios for a Danish adapted plant-rich diet were calculated, covering different food combinations and age groups. It was concluded that a whole grain intake between 75 and 100 g/10 MJ/day and, possibly more, is appropriate for age groups 2–65+ years and would not compromise micronutrient intakes (43). As dietary habits generally develop from early age, starting healthy habits early is important. Another argument for having parallel recommendations for children and adults is the social function of eating, which will be strengthened when children and adults eat the same food (44).

There are some data gaps and limitations that should be mentioned. Firstly, there is much more information available for whole grain than for refined grains, and the focus on whole grains excludes some refined cereal products such as cakes and some baked goods. However, cakes and baked goods are normally smaller contributors to the intake of grains. We did not search specifically for different sub-groups of whole grain products (e.g. bread, breakfast cereals), and thus some studies on sub-groups might have been missed. Meta-analyses including both whole grains and refined grains generally indicate more beneficial associations for whole grains/whole grain products than for refined grains/refined grain products for most health outcomes, including cardiovascular disease and mortality, diabetes type 2, and overweight/obesity (38, 45–50). Thus, even with some limitations and less certainty on findings related to refined grains, it is reasonable to recommend a high intake of whole grains partly at the expense of refined grains but also at the expense of other food groups where the intake is higher than recommended (43).

Secondly, papers analysing how substitution of refined grains with whole grains influences health outcomes are sparse. Thirdly, as noted in the overview paper of diet in the Nordic and Baltic countries (29), it would be helpful to harmonize the definition of whole grain foods, food groups, and reporting of for example whole grains in dietary surveys, and possibly also the methodology, in order to improve comparison of intakes between countries. Differences in exposure measurement could lead to uncertainty in the relationship with outcomes, particularly when pooling data. Fourthly, most observational studies are on overall cereals/whole grains, and information about associations between specific cereals species or grain products and health outcomes is warranted. There is only a handful of studies on cardiovascular diseases, cancer, and all-cause mortality, for example rice, rye, oat/oatmeal, bread, and breakfast cereals (2). Fifthly, more high-quality studies are needed. The duration of trials is generally short, and longer lasting trials would increase the strength of the evidence beyond biomarkers for disease. Many observational studies were not originally designed to measure whole grain intake, and post hoc classifications may lack precision. In addition, in observational studies, even though measures are taken to adjust for possible confounders, it is difficult to remove confounders completely. Sixthly, studies in more diverse populations are warranted. Studies, specifically on children, the elderly, pregnant and lactating women are largely missing. For dietary surveillance in the Nordic and Baltic countries, studies in immigrant populations would also be of interest.

## Integration

The substantial inverse associations between whole grains and a range of chronic diseases presented above are supported by results from randomized trials on biomarkers for disease that are likely mediators of the effects of presented associations. Meta-analyses show that intake levels in the range of 3 to 7 servings per day, equivalent of 90 to 210 g of whole grain products (or fresh weight whole grains), are associated with the lowest risks of all-cause mortality, coronary heart disease, type 2 diabetes, and colorectal cancer (24). This would be equivalent of 30 to 70 g of dry whole grains (e.g. whole grain flour or dry oatmeal). For populations eating typical western diets, increasing the intake of whole grains could contribute substantially to longevity (51). This supports a recommendation of ensuring a high intake of whole grain foods. This could also contribute to prevention of chronic diseases and to weight loss (24, 40), even though grains and grain products are important contributors to the total energy and salt intake. Hence, dietary guidelines for increased consumption of whole grains could reduce morbidity and mortality in the Nordic and Baltic countries.

## Conflict of interest and funding

The authors have not received any funding or benefits from industry to conduct this study, and report no conflicts of interests.

The authors received a small reimbursement from the Norwegian Directorate of Health for work associated with this article.
